# Disposable Colonoscopes: Unpacking the Infection‐Free Endoscopy With the REAL Price Tag—What About Performance?

**DOI:** 10.1111/den.15083

**Published:** 2025-07-04

**Authors:** Yoshihiro Kishida, Shiro Oka

**Affiliations:** ^1^ Department of Gastroenterology Hiroshima University Hospital Hiroshima Japan

1

Colonoscopy remains a fundamental procedure in the screening, diagnosis, and therapeutic management of colorectal diseases. Despite significant improvements in reprocessing protocols, the use of reusable endoscopes continues to carry an inherent risk of cross‐contamination and infection transmission [1, 2]. Although colonoscopy is generally regarded as a low‐risk procedure for infection, studies have reported colonoscopy‐associated infection rates of up to 1.6 per 1000 procedures, depending on definitions and settings [1]. This concern was brought to the forefront by reports of persistent contamination in endoscopes even after meticulous cleaning and strict adherence to reprocessing protocols [2]. These findings have prompted regulatory agencies and professional societies to reconsider reprocessing protocols and explore alternatives, including single‐use components [3]. While single‐use gastroscopes and duodenoscopes have demonstrated comparable performance to reusable counterparts in recent reports [4, 5], their colonoscopic counterparts have remained less explored in both clinical practice and the literature.

In this issue of *Digestive Endoscopy*, Wang et al. present a pilot randomized controlled noninferiority trial comparing a novel disposable colonoscope approved by both the FDA and Conformité Européenne (CE) to a conventional reusable model in patients undergoing routine colonoscopy [6]. This multicenter study enrolled 116 patients and used successful completion of colonoscopy, defined as cecal intubation with sufficient withdrawal observation, as the primary endpoint. Secondary endpoints included procedural metrics such as insertion time, polyp detection rate (PDR), adenoma detection rate (ADR), image quality, and adverse events. Notably, both groups achieved 100% success in cecal intubation. Although the disposable scope group demonstrated slightly inferior image quality, inferior operation flexibility, longer insertion times, and somewhat lower PDR and ADR, these differences were not clinically prohibitive. Importantly, most combined scores for image quality and image discernibility were rated 4 or above, and technical maneuverability scores generally ranged from 2 to 3, indicating satisfactory operability. Furthermore, the withdrawal time was comparable between the two groups. Endoscopists adapted to the disposable device after approximately 10 cases, reflecting a rapid learning curve.

The findings presented by Wang et al. have several clinical implications. First, they provide evidence that disposable colonoscopes can achieve comparable outcomes to conventional reusable scopes in terms of procedural success and diagnostic performance. This is particularly relevant in clinical environments where infection prevention is paramount, such as in immunocompromised populations, transplant units, or during outbreaks of multidrug resistant organisms [2]. Second, the observation that endoscopists became proficient after just 10 procedures suggests that the adoption curve may be less steep than initially expected, supporting the feasibility of implementation in general practice settings.

Despite these encouraging findings, several practical limitations must be acknowledged before advocating widespread adoption. One of the most frequently cited drawbacks of disposable colonoscopes is their image resolution and scope flexibility, which, although acceptable in this study, may still fall short in cases requiring meticulous mucosal visualization or procedures involving complex and precise endoscopic maneuvers. This could restrict their use in advanced diagnostic settings or therapeutic procedures such as EMR or ESD. Furthermore, insertion time was significantly longer in the disposable group, raising questions about efficiency and patient comfort during routine screening colonoscopies.

In addition, the cost disparity between reusable and disposable devices remains a formidable challenge. In this study, the per‐procedure cost of disposable colonoscopy was reported as $700–$900 [6], while reusable systems, when amortized over a high procedural volume, ranged from $188 to $500 [7]. This stark contrast raises questions about sustainability in both financial and environmental terms, particularly in countries with limited healthcare budgets. Without targeted subsidies, value‐based reimbursement models, or economies of scale, routine adoption may not be economically viable. However, some modeling studies have suggested that disposable colonoscopes may be cost‐effective in low‐volume institutions where the infrastructure and labor costs associated with reprocessing are relatively higher [7].

In parallel, the environmental footprint of disposable colonoscopes has sparked concern among clinicians and policy‐makers. Each procedure generates more than 1 kg of plastic and electronic waste, presenting a dilemma in an era increasingly focused on healthcare sustainability [8]. Innovation in recyclable or biodegradable materials, coupled with take‐back programs and circular economy models, will be crucial in addressing these environmental burdens. Collaboration between manufacturers, regulators, and environmental agencies will be necessary to minimize the ecological impact without compromising patient safety [9].

From a policy and regulatory standpoint, there is growing global interest in updating infection prevention guidelines to reflect the advent of single‐use endoscopic devices. The FDA has already issued safety communications recommending a shift toward disposable components where feasible [3]. While disposable endoscopes are widely presumed to mitigate infection risk by obviating the need for reprocessing, robust clinical evidence confirming a definitive reduction in infection rates remains limited. Meanwhile, societies such as the European Society of Gastrointestinal Endoscopy (ESGE) and the American Society for Gastrointestinal Endoscopy (ASGE) have continued to emphasize the importance of high‐level disinfection while also acknowledging the potential role of single‐use devices in infection prevention. The ASGE summit held in 2019 highlighted the promise of such innovations, though widespread adoption remains contingent upon further evaluation of cost‐effectiveness, environmental impact, and clinical performance [10]. Notably, the Japanese Gastroenterological Endoscopy Society (JGES), the official society behind *Digestive Endoscopy*, like ESGE and ASGE, is expected to continue exploring appropriate guidance for this evolving area. As more data become available, a coordinated effort among international societies will be essential to harmonize best practices.

One important consideration when interpreting current findings is that the comparator in the present trial was the Olympus CF‐HQ290, a previous‐generation reusable colonoscope. Therefore, performance differences between this model and more recent high‐definition systems should be taken into account when generalizing the results. Additionally, to support broader integration of disposable colonoscopes, future research should go beyond pilot feasibility and address long‐term effectiveness and cost–benefit profiles. Multicenter, randomized controlled trials with sufficient power should evaluate endpoints such as ADR, detection of serrated lesions, therapeutic efficacy, patient‐reported outcomes, and postcolonoscopy colorectal cancer rates. Special attention should also be paid to stratifying outcomes by endoscopist experience and institutional setting, as learning curves and resource constraints may significantly influence performance and adoption. Studies comparing outcomes in real‐world emergency or pandemic conditions would also be valuable, given the unique advantages of portability and readiness offered by single‐use scopes.

In conclusion, Wang et al. provide timely and important evidence that disposable colonoscopes can serve as a safe and effective alternative to conventional reusable scopes in selected scenarios. While not yet positioned to replace reusables entirely, their integration into clinical workflows particularly in high‐risk, low‐resource, or infection‐prone settings deserves serious consideration. Further clinical validation, thoughtful policy‐making, and environmentally responsible innovation will be essential to define the long‐term role of disposable colonoscopy in modern endoscopic practice.

## Author Contributions

Y.K.: writing – original draft preparation. S.O.: writing – review and editing. All authors approved the final version of the manuscript.

## Conflicts of Interest

The authors declare no conflicts of interest.

## Linked Articles

X. L. Wang, B. J. Li, H. W. Ye, et al. Performance of a Disposable Colonoscope for Routine Examination: Pilot Randomized Controlled Noninferiority Trial (With Video). https://doi.org/10.1111/den.15040
